# Habitat Selection and Temporal Abundance Fluctuations of Demersal Cartilaginous Species in the Aegean Sea (Eastern Mediterranean)

**DOI:** 10.1371/journal.pone.0035474

**Published:** 2012-04-20

**Authors:** Christos D. Maravelias, George Tserpes, Maria Pantazi, Panagiota Peristeraki

**Affiliations:** 1 Hellenic Centre for Marine Research, Anavyssos, Attica, Greece; 2 Hellenic Centre for Marine Research, Iraklion, Crete, Greece; Aristotle University of Thessaloniki, Greece

## Abstract

Predicting the occurrence of keystone top predators in a multispecies marine environment, such as the Mediterranean Sea, can be of considerable value to the long-term sustainable development of the fishing industry and to the protection of biodiversity. We analysed fisheries independent scientific bottom trawl survey data of two of the most abundant cartilaginous fish species (*Scyliorhinus canicula*, *Raja clavata*) in the Aegean Sea covering an 11-year sampling period. The current findings revealed a declining trend in *R. clavata* and *S. canicula* abundance from the late ′90 s until 2004. Habitats with the higher probability of finding cartilaginous fish present were those located in intermediate waters (depth: 200–400 m). The present results also indicated a preferential species' clustering in specific geographic and bathymetric regions of the Aegean Sea. Depth appeared to be one of the key determining factors for the selection of habitats for all species examined. With cartilaginous fish species being among the more biologically sensitive fish species taken in European marine fisheries, our findings, which are based on a standardized scientific survey, can contribute to the rational exploitation and management of their stocks by providing important information on temporal abundance trends and habitat preferences.

## Introduction

Cartilaginous fish constitute an ancient conservative taxonomic group that was very abundant in the world oceans. Their abundance has progressively declined over time worldwide and many species are considered vulnerable or endangered [1]–[5]. Due to their K-selection life-history traits (long life spans, slow growth, low fecundity and late attainment of sexual maturity) they are susceptible to over-exploitation and to changes in the ecosystem. The bottom trawling is considered responsible for a large amount of by-catch of cartilaginous fish throughout the world [6]. As a result cartilaginous fishes can be used as biological indicators [7] and their study and monitoring is considered vital for the conservation of the marine ecosystem.

In the Mediterranean Sea, the majority of the cartilaginous fish species are demersal and large amounts of them (over 10,000 tons/year) are caught incidentally by bottom trawlers [8]. The species most frequently caught with this gear, depending on depth and areas [9]–[12] are: *Galeus melastomus, Scyliorhinus canicula, Etmopterus spinax, Raja clavata, Raja asterias* and *Squalus acanthias*. In the Aegean Sea which is considered by the General Fisheries Council for the Mediterranean of the Food and Agriculture Organization (GFCM/FAO) as an independent management unit, very few studies on the abundance and distribution of demersal elasmobranch species have been conducted [13]–[15]. In our study we analyse an 11-year data time series of the most common demersal cartilaginous species collected from a fisheries independent scientific bottom trawl survey (The Mediterranean International Trawl Survey (MEDITS) carried out in several parts of the Mediterranean Sea since 1994 [9], [16] that covers the whole Aegean Sea (GFCM Geographical Sub l Area 22). We modeled the abundance data as functions of spatial and temporal variables, in order to identify spatio-temporal trends and built abundance density maps demonstrating on a quantitative basis the distribution of four of the most abundant cartilaginous fish species in the Aegean Sea: *Scyliorhinus canicula* (Linnaeus, 1758) (smallspotted catshark) and *Raja clavata* (Linnaeus, 1758) (thornback ray), representing more than 74% of the total demersal elasmobranch abundance caught during the period 1998–2008. (Table 1). In addition, the overall abundance of cartilaginous species is modeled.

**Table 1:** Percentage (%) contribution of the examined species to the total elasmobranchs abundance for the studied period 1998–2008.

Species	Percentage of total abundance
*R. clavata*	14.4
*S. canicula*	60.3

## Methods

### Sampling and study area

**Figure 1:**
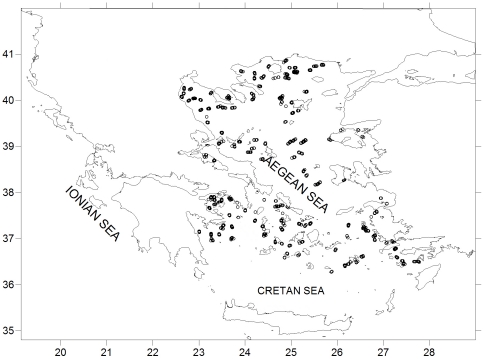
Map of the study area (Aegean Sea) indicating the sampled stations.

The MEDITS surveys are carried out annually since 1994 during the spring/early summer period. The surveys are performed in various areas of the Mediterranean Sea and include sampling at predefined stations over the shelves and the upper slopes from 10 to 800 m depth. Further details on the sampling protocol can be found elsewhere [9], [16]. Since 1996, the sampling scheme of the Greek MEDITS survey is consistent covering annually (with the exception of 2002 and 2007, when the survey was not accomplished) a total of about 100 stations in the Aegean Sea (Fig. 1). No specific permits were required for the described field studies.

In the present study, due to data accessibility we focused our analysis on abundance data from the surveys carried out in the 1998–2008 period in the Aegean Sea. Abundance was expressed in terms of number per square km of swept area (n/km^2^). Analysis included the following species: *S. canicula* (smallspotted catshark) and *R. clavata* (thornback ray).. Apart from the above, the overall abundance of cartilaginous species was modeled including also the species *Squalus acanthias, Squalus blainville*, *Galeus melastomus, Mustelus mustelus, Raja asterias* and *Torpedo marmorata*.

### Data analysis

The analysis utilized generalized additive modeling (GAMs) techniques [17], to investigate the effects of station position and depth on species abundance. The main advantage of GAMs over traditional regression methods are their capability to model non-linear relationships (a common feature of many ecological datasets) between a response variable and multiple explanatory variables using non-parametric smoothers. In the present case the non-linear predictors included sampling position (entered as the latitude - longitude interaction) and depth, while the “year” was entered as a categorical variable.

A two-stage modeling approach was implemented which allows dealing with the large number of zeros encountered in the species abundance matrix. The first stage of this technique models the probability of presence of each species, and the second stage the corresponding relative abundance, considering only the stations with positive observations. In this way, overall relationships are not masked by the large number of zero values (i.e., conditioned on presence) [18]. In the first stage a GAM with binomial error structure and logit link was applied. In the second stage, the choice of the most appropriate link function and error distribution for the analysis of the non-zero abundance rates was made on the basis of residual plots. Preliminary analysis showed that a Gamma model accompanied by a log link function was the most appropriate for the current data sets. In all cases, the smooth function was represented using cubic splines, estimated by penalized iterative least squares [19]. Model fitting was accomplished using the mgcv package [19], [20] under the R language environment [21]. The predicted abundance values from the GAM analysis were used to construct density distribution maps for the studied species in the Aegean Sea. Maps were generated using the SURFER software [22] and interpolation was made by means of the “inverse distance to a power” gridding method [23].

## Results

**Figure 2:**
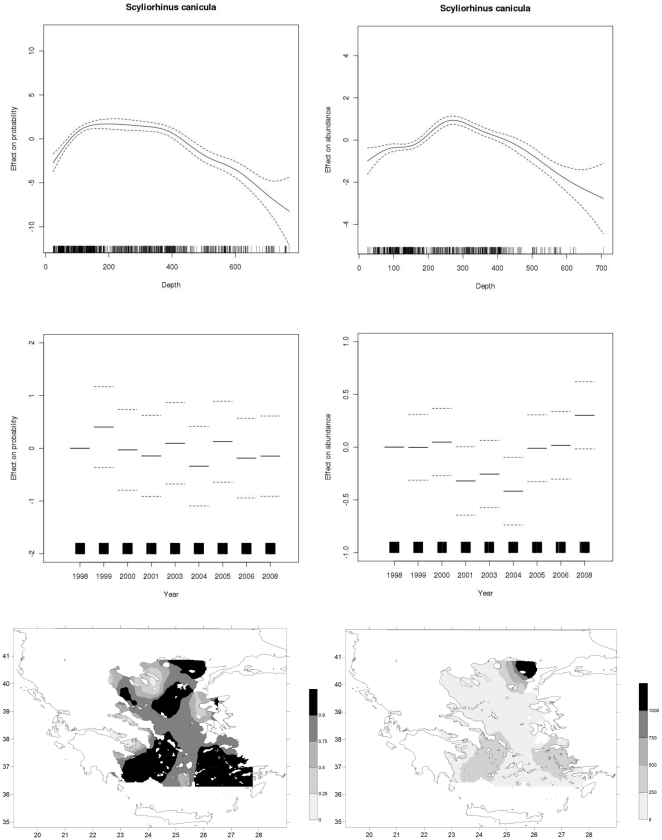
GAM derived effects of depth and year and distribution maps for *S. canicula* based on the presence/absence information (left hand column) and abundance data (right hand column).

**Figure 3:**
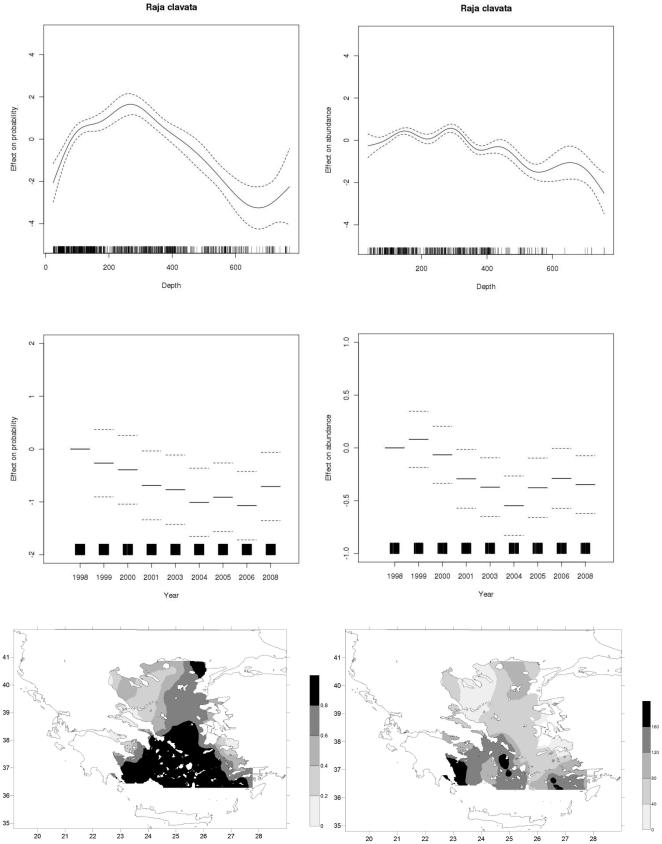
GAM derived effects of depth and year and distribution maps for *R. clavata* based on the presence/absence information (left hand column) and abundance data (right hand column).

**Figure 4:**
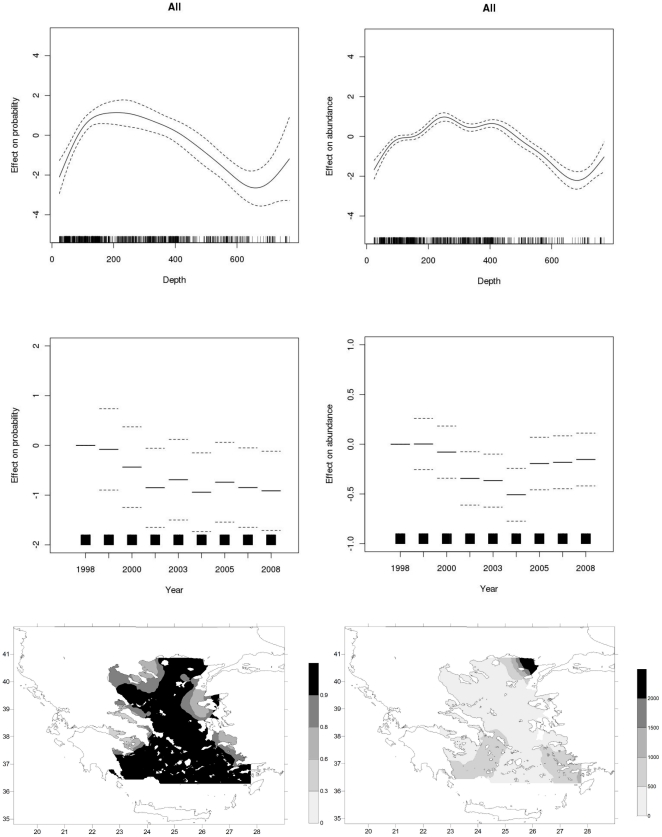
GAM derived effects of depth and year and distribution maps for all cartilaginous species based on the presence/absence information (left hand column) and abundance data (right hand column).

The analysis of deviance of the applied GAMs (not shown here) indicated that all explanatory variables were significant. The explained deviance was greater than 45% in most of the cases, suggesting that the models have a relatively high explanatory power and predictability (Table 2). Figures 2–4 show the effect (smoother plot) of the depth and year predictors on abundance, or probability of presence in the case of the binomial model, for different values of the predictor (x-axis). The zero line indicates mean model estimates, while the y-axis is a relative scale where the effect of different values of the predictors on the response variable is shown. Hence, negative values on the y-axis indicate that at the corresponding levels of the predictor (x-axis), the model estimates abundance/probability that is lower than the mean, while the opposite holds for positive values on the y-axis. Findings by species are given below.

**Table 2:** Deviance explained by the applied GAM models.

Species	Model type	Deviance explained (%)
Total abundance	Binomial	34.8
	Gamma	49.8
*S. canicula*	BinomialGamma	50.647.1
*R. clavata*	BinomialGamma	35.347.3

### *S. canicula* (Smallspotted catshark)

The species has a wide geographic distribution pattern with preference for depths between 200–400 m (Fig. 2). The species density is higher in certain regions at the Aegean Sea that are within this depth-range. Although there are annual variations in presence/absence probability rates, no overall specific trend is evident. Abundance rates however, are relatively low in the 2001–2004 period and are increasing after wards, reaching a maximum in 2008.

### *R. clavata* (Thornback ray)

Results indicated that the species is more abundant in the southern part of the Aegean basin showing preference for depths ranging from 200–380 m (Fig. 3). Both probability of presence and abundance rates show a declining trend till 2004 and are recovering since 2005, not reaching however, the levels of late 90's.

### Total abundance

Total abundance of the cartilaginous fish species in the Aegean Sea, demonstrated a decreasing trend up to 2004 and substantially stable values afterwards (Fig. 4). Species have a wide geographical and bathymetric distribution with specimens found up to 700 m, although higher abundance occurs in depths ranging from ca 180–430 m. Concerning their geographical distribution, the species demonstrated higher abundances in the north-eastern part, as well as, in specific regions of the southern Aegean Sea.

## Discussion

The use of statistical models to predict the likely occurrence or distribution of fish species is becoming an increasingly important tool in conservation planning and fisheries management. Results from the present work suggested a relationship between cartilaginous fish dynamics and spatial factors. *R. clavata* were found more abundant in the southern part of the Aegean Sea, while *S. canicula* was found more abundant in the N. Aegean (Thracian Sea). Depth appeared to be one of the key determining factors for the selection of habitats for all species that were included in the study, along with the total abundance of cartilaginous fish.

In particular, results indicated that habitats with the higher probability of finding cartilaginous fish species were those located in intermediate waters (depth: 200–400 m), which is in agreement with previous works in the area [14]. Species' clustering was found to be preferentially higher in regions of the southern Aegean Sea.

The observed spatial patterns could be attributed to the specific topographic and bathymetric conditions of the Aegean Sea. For instance, the northern Aegean Sea is characterized by an extended continental shelf; smooth muddy/sandy bottoms and higher nutrient concentrations, whereas the southern Aegean Sea is characterized by a narrow continental shelf and predominance of great, steep depths [24]. The distribution of the species, however, is a complex phenomenon, which is related to various abiotic and biological factors. Additional information on currents, temperature, salinity etc. are important and ought to be taken into account in future studies. Due to our limited understanding of the dynamic ecology of these species, it is regarded necessary to consider all possible causes for the observed distributional pattern, including fishery.

In general, the abundance of the examined species showed a declining trend from the late ′90 s until 2004 and this is more evident in the case of *R. clavata*. Given that the examined species and particularly *R. clavata* show preference for depths up to 300 m it can be speculated that this decline is, at least partially, due to the trawling fishing pressure, which is generally more intense at depths between 50–300 m [13]. Although specific selectivity studies have not been carried out, it can be hypothesized that increases in trawl-mesh size enforced in early 2000's may have contributed to the abundance recoveries observed after 2004. This is supported by observations on-board commercial bottom trawlers, indicating that the minimum size of captured individuals of *R. clavata* and *S. canicula* in the period 2003–2006 was higher than that observed in the years 1995–2000 [15]. For both species, the same authors have also reported statistically significant differences among mean sizes for the aforementioned periods. The higher mean values have been recorded in the years 2003–2006.

Although cartilaginous fish species are among the more biologically sensitive fish species taken in European marine fisheries, their stocks are in most cases poorly studied. Few studies accomplished in certain central and western Mediterranean areas indicate declining population trends [25], [7], [2]. In the eastern Mediterranean, Damalas and Vassilopoulou (2011) who studied temporal variations in chondrichtyan catch rates of commercial fisheries in a limited area of the Aegean Sea, are reporting an overall abundance decline for the 1995–2006 period, although they mention that such a finding is not valid for several of the species they examined. Their sampling scheme, however, apart from the small area-coverage, does not include important species habitats as it covers a rather narrow depth-range (50–339 m).

Increasing focus on global and regional patterns of biodiversity necessitates reliable models of species presence/absence. In a multispecies environment, such as in the Mediterranean Sea, binary classification methods of ecologically important demersal fish, based on commonly measured quantitative abiotic factors, are of major significance due to the role certain fish species play in the marine food web. Given the high position of most cartilaginous in the food-chain, their management needs particular attention, above all in order to maintain biodiversity and ecosystem structures. Management measures should pay consideration to their long life span and late maturation. The results of the current study, although are not directly informing about the state of the cartilaginous fish stocks in the Aegean Sea, they can assist their assessment and management by revealing temporal abundance trends that are based on a standardized scientific survey. Likewise, the identification of reliable habitat models of common cartilaginous species may help conservation planning by reducing by-catch of non-target species; thus, protection of biodiversity may be an added benefit. Evidently, such habitat models could be useful tools for decision-making and conservation planning.
